# Recurrent Life-Threatening Gastrointestinal Bleeding From Primary Intestinal Choriocarcinoma: A Case Report

**DOI:** 10.7759/cureus.41823

**Published:** 2023-07-13

**Authors:** Houssem Ammar, Mohamed Amine Said, Mahdi Beltaifa, Marwa Azri, Rym Zgaya, Wiem Majdoub, Oussama Belkacem, Rahul Gupta, Habiba Ben Hamada, Ali Ben Ali

**Affiliations:** 1 General Surgery, Sahloul Hospital, University of Sousse, Sousse, TUN; 2 Obstetrics and Gynaecology, Farhat Hached Hospital, University of Sousse, Sousse, TUN; 3 Pathology, Sahloul Hospital, University of Sousse, Sousse, TUN; 4 Gastrointestinal Surgery, Synergy Institute of Medical Sciences, Dehradun, IND; 5 Anaesthesiology, Sahloul Hospital, University of Sousse, Sousse, TUN

**Keywords:** choriocarcinoma, rebleeding, jejunal choriocarcinoma, beta-human chorionic gonadotropin (β-hcg), human chorionic gonadotrophin, oncology, chemotherapy, surgery, intestinal bleeding, primary choriocarcinoma

## Abstract

Choriocarcinoma is a highly aggressive, malignant tumor that arises from trophoblastic cells. Although choriocarcinomas usually arise in the genital organs, they can also originate in extragenital organs, but gastrointestinal tract lesions are rare. Gastrointestinal choriocarcinoma can be primary or metastatic. Most primary gastrointestinal choriocarcinomas are associated with adenocarcinomas.

We report a case of jejunal choriocarcinoma presenting with acute abdominal pain and intestinal bleeding. The patient had a very high serum beta-human chorionic gonadotropin (β-HCG) level with an isolated jejunal lesion on contrast-enhanced computed tomography of the abdomen and pelvis. The patient underwent emergency surgical resection of the jejunal lesion with good recovery. The histopathological analysis of the resected specimen confirmed the diagnosis of choriocarcinoma. However, the patient suffered from life-threatening rebleeding one month after surgery and succumbed to her illness.

## Introduction

Choriocarcinoma (CC) is a highly aggressive, malignant tumor that usually originates from the trophoblastic tissue in women and the germ cells of the gonads in men. It is typically observed in women after molar, normal, or ectopic pregnancy and abortion [1,2]. The incidence of CC is about 9.2 in 40,000 pregnancies in Southeast Asia [3]. CC can be gestational or non-gestational. CC has the unique characteristic of early hematogenous metastases to the lungs, kidneys, central nervous system, liver, and gastrointestinal tract [4]. The gastrointestinal tract is a rare site for metastases, with a frequency of less than 5% [5]. The most common site of gastrointestinal lesions is the stomach [6,7,8].

Extragenital primary CC of the gastrointestinal tract is even rarer, and very few cases have been reported in the English literature [4,6,8]. Due to high vascularity, most cases of gastrointestinal CC present with bleeding [8]. The characteristic feature of CC is the elevation of serum beta-human chorionic gonadotropin (β-HCG) levels. The gastrointestinal lesions are visible on abdominal imaging such as contrast-enhanced computed tomography (CECT) of the abdomen. Here, we report a case of primary jejunal CC presenting with acute abdominal pain and gastrointestinal bleeding in a female patient who continued to have elevated serum β-HCG levels despite surgical resection of the jejunal lesion.

## Case presentation

A 39-year-old woman, gravida 2, para 2, was admitted to the emergency room for headaches, acute abdominal pain, and melena for the past two months. She had no history of abnormal vaginal bleeding. On physical examination, she was hemodynamically stable. The abdominal examination was unremarkable. A rectal examination confirmed melena. Per vaginal examination, the cervix and vaginal wall were unremarkable. Blood investigations at admission showed severe anemia (a hemoglobin level of 5 g/dl). Her platelet count was 2.05 lac/mm3, and her coagulation profile was within normal limits. Her serum beta human chorionic growth hormone (β-HCG) level was very high (200 000 mUI/mL). The rest of the blood tests were normal. The transabdominal and transvaginal ultrasounds did not indicate any intrauterine or extrauterine pregnancy or any other gynecological pathology to explain the reason for the high β-HCG. She was resuscitated with a blood transfusion and intravenous fluids. Upper and lower gastrointestinal endoscopies did not identify any sites of recent bleeding.

**Figure 1:**
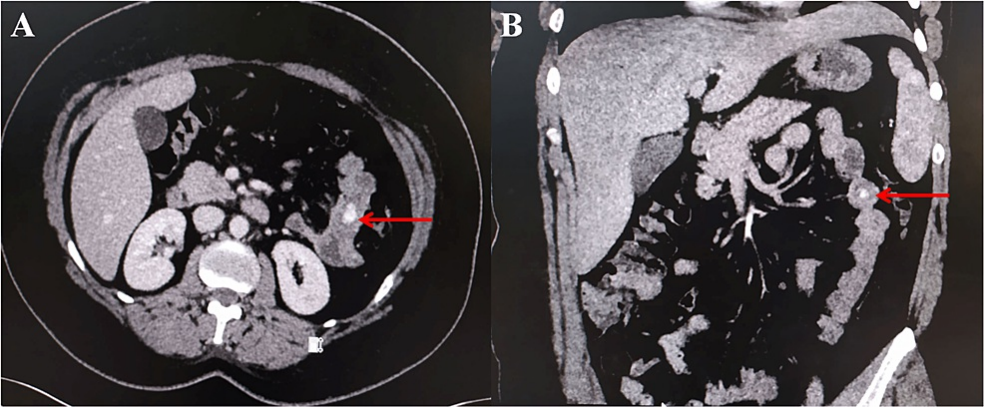
Contrast-enhanced computed tomography shows intraluminal extravasation of the intravenous contrast in the jejunum (red arrow) on axial and coronal sections (A, B).

Contrast-enhanced computed tomography (CECT) of the abdomen revealed intraluminal extravasation of the intravenous contrast in the jejunal loops without any obvious neoplastic pathology or genital lesion (Figure 1).

**Figure 2:**
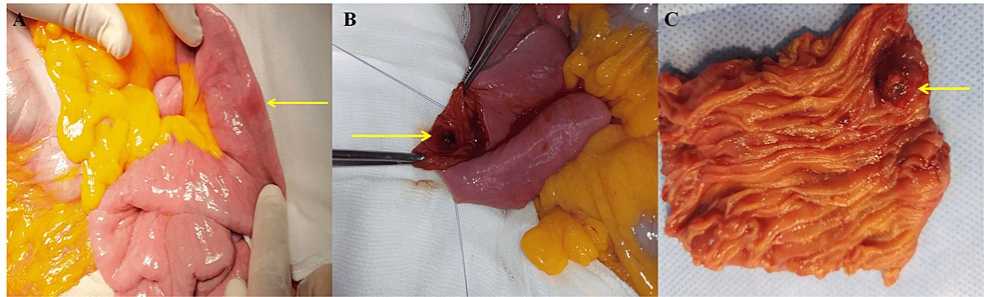
Intraoperative photograph showing the endoluminal jejunal lesion (A), which was found to be actively bleeding (B), and a segmental jejunal resection showing a small endoluminal lesion (C).

The patient underwent an emergency laparotomy. On abdominal exploration, a small endoluminal lesion was palpable in the jejunum, located 30 cm from the Treitz ligament. After enterotomy, the lesion was found to be actively bleeding (Figure 2).

There was no macroscopic evidence of peritoneal dissemination or liver metastasis, and the palpation of the rest of the jejunum, ileum, and colon was normal. A segmental jejunal resection, including the lesion, with end-to-end anastomosis was performed.

**Figure 3:**
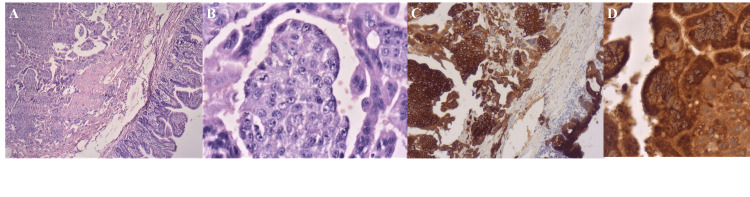
Microscopic examination revealed a biphasic pattern consisting of syncytiotrophoblast cells intermingled with mononuclear cytotrophoblast cells (hematoxylin and eosin (HE) X40) (A). On high magnification, the tumor is composed of multinucleated syncytiotrophoblast cells and mononuclear cytotrophoblasts with occasional mitotic figures and marked cytological atypia (HE X400) (B). Immunohistochemical study showing tumor cells are diffusely and strongly positive for human chorionic gonadotropin and cytokeratin (C, D).

On microscopic examination, a tumor was found in the submucosa layer of the jejunum with a biphasic pattern consisting of syncytiotrophoblast cells intermingled with mononuclear cytotrophoblast cells. On higher magnification, the tumor was composed of multinucleated syncytiotrophoblast cells and mononuclear cytotrophoblasts with occasional mitotic figures and marked cytological atypia. Tumor cells were diffusely and strongly positive for hCG and cytokeratin (CK) on immunological staining (Figure 3).

The final diagnosis of CC of the jejunum was made.

The postoperative course was uneventful, with the passage of normal colored stools and stabilization of the hemoglobin levels; however, the β-HCG levels remained high. In order to detect the primary site of the tumor, various imaging tests, including thoracoabdominal CT and pelvic magnetic resonance imaging (MRI), were performed, and no other sites of CC could be detected.

Chemotherapy with etoposide, methotrexate, and actinomycin D was started four weeks postoperatively. However, during her first course of chemotherapy, she presented with melena and then anal bleeding, requiring hospitalization in the intensive care unit with a blood transfusion. An abdominal CT and red blood cell scintigraphy were urgently performed but failed to identify the origin of the bleeding. Coagulation profile tests could not be done. The patient's clinical condition rapidly deteriorated within a few minutes, and she developed cardiovascular collapse. Consequently, immediate resuscitation measures were initiated to stabilize the patient's condition. However, despite the efforts made, the patient died following a cardiac arrest. An autopsy was requested, but the patient's family did not give consent.

## Discussion

Choriocarcinoma (CC) is a rare and highly malignant neoplasm. It most commonly arises from the trophoblastic tissue in the background of molar pregnancy, abortion, ectopic pregnancy, and normal delivery [2]. However, cases of CC of extragenital origin have been documented [2,4,6,8]. The gastrointestinal tract is a rare site of origin for CC, and the stomach is the most common part of the gastrointestinal system to be affected by it [6,8]. 

The exact pathogenesis of primary extragenital CC is not known. However, primary gastric and colorectal CC has been associated with adenocarcinoma in several reported cases [8, 9]. McKechnie and Fechner suggested that CC is the result of the dedifferentiation of adenocarcinoma cells to the level of the embryonal ectoderm [7].

In a pooled analysis of 53 cases of gastric CC, there were 37 males and 16 females, with a mean age of 62.4 years [8]. The most frequent site of gastric CC was the lower third of the stomach, similar to that observed in gastric adenocarcinoma. Among these 53 cases, 70% had an adenocarcinoma component. Also, most of the patients had metastatic disease [8].

Intestinal CC is very rare, with less than 30 cases reported in the English literature to date, with most of them being metastatic [10]. Clinically, the determination of the primary or secondary nature of extragenital CC is difficult as the primary lesion may not be visible in the current presentation. Hence, it is important to exclude the past history of molar pregnancy or primary uterine CC by detailed clinical history since it has been described in the literature that uterine CC can spontaneously regress from its primary localization before the appearance of metastatic lesions. Indeed, the difficulty in determining whether the tumor is primary or secondary has been widely discussed in the literature, and many authors have been unable to determine it [8].

The preoperative diagnosis of primary extragenital CC can be challenging. It often has an unusual presentation, but the most frequent symptoms of intestinal CC, whether primary or secondary, are gastrointestinal bleeding (hematemesis or melena) and severe anemia, as seen in the present case [2,4,6,8]. Other less common symptoms include abdominal pain and constipation due to intestinal obstruction or perforation [2,10]. 

Elevated serum β-HCG levels are the hallmark of CC. Serum β-HCG is a simple, non-expensive, non-invasive, and widely available test that can guide the diagnosis and treatment of CC. Serum β-HCG higher than 100,000 mIU/mmL in the absence of normal or molar pregnancy is highly suggestive of CC [11]. However, the final diagnosis is made by histology, as β-HCG can be secreted by other tumors [12].

Contrast-enhanced computed tomography (CECT) of the abdomen and pelvis is the imaging modality of choice for the detection of intestinal CC. They appear as intestinal wall thickening or a soft tissue shadow with contrast enhancement [10]. There may be contrast leakage in cases of active bleeding. In the present case, only one jejunal lesion was visible on the CECT of the abdomen, which was excised. However, the serum β-HCG levels continued to remain high, and the patient suffered from rebleeding and succumbed to her illness. This indicates that probably some lesions were missed on the CECT of the abdomen, which led to rebleeding.

Endoscopy can be used in the diagnosis of gastrointestinal CC and often provides access to the tumor for histological evidence. However, it has been reported that histological diagnosis by small biopsy is difficult and has a low diagnostic yield [8]. Additionally, a biopsy carries the risk of life-threatening bleeding as CC is a vascular tumor. Hence, the European Medical Oncology Society has recommended that chemotherapy can be started in suspected cases even without a biopsy [13].

Other diagnostic modalities described for the diagnosis of choriocarcinoma include human placental lactogen (hPL) and placental alkaline phosphatase (PLAP) [14]. Cytogenetic studies can be used to identify DNA polymorphisms in the tumor tissues, which can help in distinguishing the gestational and non-gestational nature of choriocarcinoma by objectifying the androgenetic nature of the tumor [15].

The treatment of intestinal CC depends on the manifestation and severity of the disease. Choriocarcinomas are highly chemo-sensitive [13]. The most commonly administered chemotherapeutic drugs include methotrexate, cyclophosphamide, and actinomycin D [13]. Emergency surgery may be necessary in cases of intestinal perforation, obstruction, or unexplained intestinal bleeding. Surgery allows oncological resection of the tumor and confirms the diagnosis by histopathological analysis of the tumor. In the present case, massive bleeding occurred during the first chemotherapy session.

The prognosis for gestational CC is very good with chemotherapy. However, the prognosis of CC patients with intestinal CC is poor, as they most often have multiple pulmonary and hepatic metastases, unlike the index case [10]. The leading cause of death in patients with metastatic CC is hepatic failure due to extensive tumor metastasis, disseminated intravascular coagulation, and hemorrhage [8].

## Conclusions

Choriocarcinoma of the small intestine is a rare and aggressive disease that often presents with gastrointestinal bleeding. Serum β-HCG levels can help in making a preoperative diagnosis. It appears as a contrast-enhancing lesion on abdominal CT. However, all lesions may not be detectable on CT, as seen in the present case. Chemotherapy is the mainstay of treatment for CC. But emergency surgery may be required to control bleeding in patients with intestinal CC. Chemotherapy for CC must be initiated as early as possible to achieve disease control.

## References

[1] Yousefi Z, Mottaghi M, Rezaei A, Ghasemian S (2016). Abnormal presentation of choriocarcinoma and literature review. Iran J Cancer Prev.

[2] Cho EB, Byun JM, Jeong DH (2016). Metastatic choriocarcinoma as initial presentation of small bowel perforation in absence of primary uterine lesion: a case report. Tumori.

[3] Ngan HY, Seckl MJ, Berkowitz RS (2018). Update on the diagnosis and management of gestational trophoblastic disease. Int J Gynaecol Obstet.

[4] Iyomasa S, Senda Y, Mizuno K (2003). Primary choriocarcinoma of the jejunum: report of a case. Surg Today.

[5] Yokoi K, Tanaka N, Furukawa K (2008). Male choriocarcinoma with metastasis to the jejunum: a case report and review of the literature. J Nippon Med Sch.

[6] Guzmán-López JC, Lino-Silva LS, Salcedo-Hernández RA, Zepeda-Najar C (2019). Report of three cases of gastric choriocarcinomas-an emphasis on morphologic changes in the non-affected gastric mucosa. J Gastrointest Oncol.

[7] McKechnie JC, Fechner RE (1971). Choriocarcinoma and adenocarcinoma of the esophagus with gonadotropin secretion. Cancer.

[8] Kobayashi A, Hasebe T, Endo Y (2005). Primary gastric choriocarcinoma: two case reports and a pooled analysis of 53 cases. Gastric Cancer.

[9] Boyce J, Tawagi K, Cole JT (2020). Primary colon adenocarcinoma with choriocarcinoma differentiation: a case report and review of the literature. J Med Case Rep.

[10] Wang Y, Wang Z, Zhu X, Wan Q, Han P, Ying J, Qian J (2022). Intestinal metastasis from choriocarcinoma: a case series and literature review. World J Surg Oncol.

[11] Menczer J, Modan M, Serr DM (1980). Prospective follow-up of patients with hydatidiform mole. Obstet Gynecol.

[12] Murhekar KM, Anuratha JN, Majhi U, Rajkumar T (2009). Expression of human chorionic gonadotropin beta in gastric carcinoma: A retrospective immunohistochemical study. Indian J Med Paediatr Oncol.

[13] Seckl MJ, Sebire NJ, Fisher RA, Golfier F, Massuger L, Sessa C (2013). Gestational trophoblastic disease: ESMO Clinical Practice Guidelines for diagnosis, treatment and follow-up. Ann Oncol.

[14] Verbeek W, Schulten HJ, Sperling M (2004). Rectal adenocarcinoma with choriocarcinomatous differentiation: clinical and genetic aspects. Hum Pathol.

[15] Fisher RA, Newlands ES, Jeffreys AJ, Boxer GM, Begent RH, Rustin GJ, Bagshawe KD (1992). Gestational and nongestational trophoblastic tumors distinguished by DNA analysis. Cancer.

